# Clinical application of targeted next-generation sequencing in severe pneumonia: a retrospective review

**DOI:** 10.1186/s13054-024-05009-8

**Published:** 2024-07-08

**Authors:** Peng Zhang, Baoyi Liu, Shuang Zhang, Xuefei Chang, Lihe Zhang, Dejian Gu, Xin Zheng, Jiaqing Chen, Saiyin Xiao, Zhentao Wu, Xuemin Cai, Mingfa Long, Wenjie Lu, Mingzhu Zheng, Rongrong Chen, Rui Gao, Yan Zheng, Jinhua Wu, Qiujuan Feng, Gang He, Yantang Chen, Weihao Zheng, Wanli Zuo, Yanming Huang, Xin Zhang

**Affiliations:** 1 Department of Critical Care Medicine, Jiangmen Central Hospital, Jiangmen, 529030 China; 2 Clinical Experimental Center, Jiangmen Engineering Technology Research Center of Clinical Biobank and Translational Research, Jiangmen Central Hospital, Jiangmen, 529030 China; 3 Department of Respiratory and Critical Care Medicine, Jiangmen Central Hospital, Jiangmen, 529030 China; 4 Geneplus-Beijing Institute, Beijing, 102206 China; 5 Department of Research and Development, Guangdong Research Institute of Genetic Diagnostic and Engineering Technologies for Thalassemia, Hybribio Limited, Guangzhou, 510000 China; 6 Department of Clinical Laboratory, Jiangmen Central Hospital, Jiangmen, 529030 China; 7 Department of Infectious Diseases, Jiangmen Central Hospital, Jiangmen, 529030 China; 8 Dongguan Key Laboratory of Medical Bioactive Molecular Developmental and Translational Research, Guangdong Provincial Key Laboratory of Medical Molecular Diagnostics, Guangdong Medical University, Dongguan, 523808 China

**Keywords:** Severe pneumonia, Targeted next-generation sequencing, Metagenomics next-generation sequencing, Culture

## Abstract

**Background:**

The precise identification of the underlying causes of infectious diseases, such as severe pneumonia, is essential, and the development of next-generation sequencing (NGS) has enhanced the effectiveness of pathogen detection. However, there is limited information on the systematic assessment of the clinical use of targeted next-generation sequencing (tNGS) in cases of severe pneumonia.

**Methods:**

A retrospective analysis was conducted on 130 patients with severe pneumonia treated in the ICU from June 2022 to June 2023. The consistency of the results of tNGS, metagenomics next-generation sequencing (mNGS), and culture with the clinical diagnosis was evaluated. Additionally, the results for pathogens detected by tNGS were compared with those of culture, mNGS, and quantitative reverse transcription PCR (RT-qPCR). To evaluate the efficacy of monitoring severe pneumonia, five patients with complicated infections were selected for tNGS microbiological surveillance. The tNGS and culture drug sensitisation results were then compared.

**Results:**

The tNGS results for the analysis of the 130 patients showed a concordance rate of over 70% with clinical diagnostic results. The detection of pathogenic microorganisms using tNGS was in agreement with the results of culture, mNGS, and RT-qPCR. Furthermore, the tNGS results for pathogens in the five patients monitored for complicated infections of severe pneumonia were consistent with the culture and imaging test results during treatment. The tNGS drug resistance results were in line with the drug sensitivity results in approximately 65% of the cases.

**Conclusions:**

The application of tNGS highlights its promise and significance in assessing the effectiveness of clinical interventions and providing guidance for anti-infection therapies for severe pneumonia.

**Supplementary Information:**

The online version contains supplementary material available at 10.1186/s13054-024-05009-8.

## Background

Pneumonia is a globally prevalent infectious disease affecting various age groups. Inadequate treatment can lead to severe pneumonia, potentially causing multiorgan failure and death [1–5]. The timely and accurate identification of pathogens is crucial for improving the chances of survival in critically ill patients. Delayed or insufficient antimicrobial therapy may result in unfavourable outcomes [6–8]. Multiple pathogenic bacterial infections are common in people with long-term medical conditions or those who have received treatment, particularly during the COVID-19 pandemic [1, 9]. Hence, identifying pathogens is crucial for treating severe pneumonia.

Culture methods are extensively used to diagnose pneumonia, offering the benefits of cost-effectiveness, quantifying the presence of pathogens, and facilitating antibiotic susceptibility testing; however, cultures can be time-consuming and less effective in detecting atypical infections [10]. Next-generation sequencing (NGS) is a rapid and comprehensive method for detecting respiratory infections. It can better detect these pathogens than traditional assays and identify them in patients who test negative in culture tests [11]. The main challenges of NGS are its cost, complexity, and standardisation [12–14].

Metagenomics next-generation sequencing (mNGS) enables the quick and unbiased identification of harmful viruses, bacteria, fungi, and parasites. It can identify severe diseases and challenging infections that warrant urgent care [13, 15, 16]. Metagenomics is anticipated to become the first-line detection method for severe pneumonia [17]. Previously, we reported the significance of mNGS in ARDS diagnosis, treatment, and prognosis [16, 18]. Nevertheless, mNGS is a costly method for simultaneously conducting DNA and RNA tests and is influenced by human genetics. It yields relatively low data readouts from the pathogen genome, making its use challenging for regular and ongoing disease monitoring [19]. Unlike mNGS, targeted next-generation sequencing (tNGS) requires the development of precise primers or probes for pre-selected pathogens for panel construction. This method is cost-effective, highly specific, requires minimal sample quantities, and can avoid influence by human DNA [20]. tNGS has been used to identify pathogens in lung, bloodstream, bone and joint, and mycobacterial infections [21–24]. It has also been used to detect genes associated with treatment resistance [25, 26], demonstrating its potential for clinical applications [27, 28]. To date, the systematic evaluation of the clinical use of tNGS for severe pneumonia has been insufficient. This retrospective study therefore assessed the use of tNGS in the clinical management of severe pneumonia.

## Materials and methods

### Case recruitment and sample collection

**Fig. 1:**
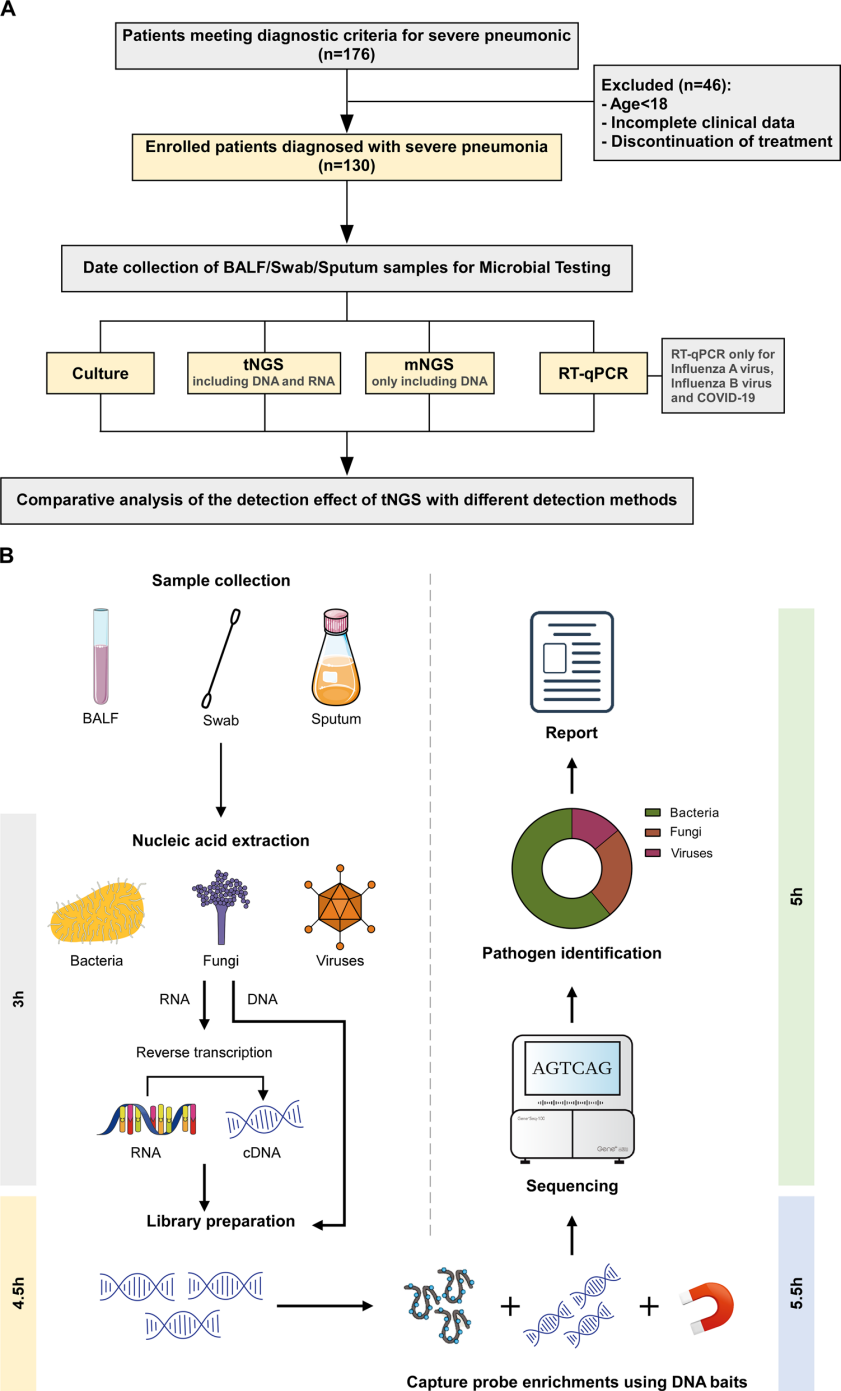
Overview of the research scheme. **A** Flowchart of the experimental design. **B** Schematic diagram of tNGS sequencing assay steps

A retrospective analysis was conducted on patients with severe pneumonia admitted to the ICU of Jiangmen Central Hospital between June 2022 and June 2023. The study protocol was approved by the Ethics Review Committee of Jiangmen Central Hospital (No: 2021-15). Patients or their legal representatives provided their signatures on an informed consent form. A total of 130 patients were identified for analysis, and their clinical data were collected (Fig. 1A). The inclusion criteria were as follows: (1) meeting the diagnostic criteria for severe pneumonia and (2) being 18 years of age or older. The exclusion criteria included (1) the premature discontinuation of treatment and (2) insufficient case data.

All the patients underwent extubation and mechanical ventilation in the ICU. Fibreoptic bronchoscopy was used to acquire bronchoalveolar lavage fluid (BALF) or sputum for microbiological testing. Pharyngeal swab samples were obtained from a subset of the patients. Upon the transfer of the patients to the ICU, we retained initial samples from patients who had received a diagnosis of severe pneumonia within 24 h. Additional samples were collected weekly for re-examination. Subsequently, the samples were sent to a clinical laboratory for microbiological analyses. The remaining specimens were sent to the clinical experimental centre and dispatched to the GenePlus-Beijing Institute (Beijing, China) for mNGS and tNGS (Fig. 1B), and Hybribio Co., Ltd. (Guangzhou, China) conducted quantitative reverse transcription PCR (RT-qPCR) analyses.

### Clinical treatment of patients with severe pneumonia

Patients received treatment based on the Chinese guidelines for diagnosing and treating community-acquired pneumonia in adults [29] and the Chinese guidelines for diagnosing hospital-acquired pneumonia and ventilator-associated pneumonia in adults [30]. These guidelines were used in combination with the clinical indicators of infection and imaging data. If the exact microbes that caused the infection could not be positively identified, empirical treatments with anti-infection drugs were administered, and antimicrobial therapy was subsequently modified based on the microbiological test results.

### Concordance evaluation

Evaluating the concordance of the tNGS, mNGS, and culture results relative to the clinical diagnoses involved a thorough assessment by three experienced clinicians holding the title of associate senior clinician or higher and having over 10 years of ICU experience. The clinicians integrated the results to reach final diagnoses. The diagnoses considered the clinical characteristics of the patients, results of standard pathogenic tests, NGS results, pathological data, imaging findings, and other relevant factors.

### NGS, culture, and RT-qPCR microbiology testing

Of 130 samples, 126 were BALF, and the remaining 4 were sputum samples (*Mycobacterium tuberculosis* infection). The detailed steps are provided in Additional file 1.

### Statistical analysis

Continuous variables that exhibited a normal distribution were represented as the mean ± standard deviation ($\overline{\text{x}}$ ± s), but those that did not conform to a normal distribution were represented as the median with the interquartile range [M (P25, P75)]. Group comparisons were conducted using unpaired *t*-tests or Mann–Whitney U tests. The frequencies and percentages [N (%)] were utilised for categorical variables, and comparisons between groups were conducted using either the χ^2^ test. The positive group identified using both methods served as the reference diagnostic criterion. A graph depicting the receiver operating characteristic (ROC) curve and area under the curve (AUC), together with a 95% confidence interval, was generated. Heatmaps were generated using the ggplot package in R or Multiple Experiment Viewer software. Data analyses were performed using GraphPad Prism 9.3 software (GraphPad Software, Inc.), R 4.3.1 software (http://www.r-project.org, The R Foundation), and Multiple Experiment Viewer software (https://sourceforge.net/projects/mev-tm4/). Differences were deemed statistically significant if *P* < 0.05 (two-sided).

## Results

### General information on the patients

A total of 130 patients with severe pneumonia were included in this study. The patient demographic information, underlying medical conditions, and specific treatments received in the ICU are presented in Table 1.

**Table 1:** Clinical characteristics of patients with severe pneumonia

Clinical characteristic	
Age, M (P25, P75)	59 (56,75)
Gender, N (%)	
Male	94 (72.3)
Female	36 (27.7)
Types of Pneumonia, N (%)	
Community acquired pneumonia	55 (42.3)
Hospital acquired pneumonia	75 (57.7)
Underlying diseases, N (%)	
Hypertension	70 (53.8)
Coronary heart disease	17 (13.1)
Chronic obstructive pulmonary disease	21 (16.2)
Chronic renal insufficiency	32 (24.6)
Diabetes	39 (30.0)
Immunosuppression	35 (26.9)
Tumors	30 (23.1)
Smoking	44 (33.8)
Excessive drinking	11 (8.4)
Special treatments in the ICU, N (%)	
Use of vasoactive drugs	51 (39.2)
CRRT	31 (23.8)
ECMO	1 (0.8)
Prone ventilation	23 (17.7)

CRRT, continuous renal replacement therapy; ECMO, extracorporeal membrane oxygenation

### Evaluation of the concordance of the three methods (tNGS, culture, and mNGS) with clinical diagnoses

**Fig. 2:**
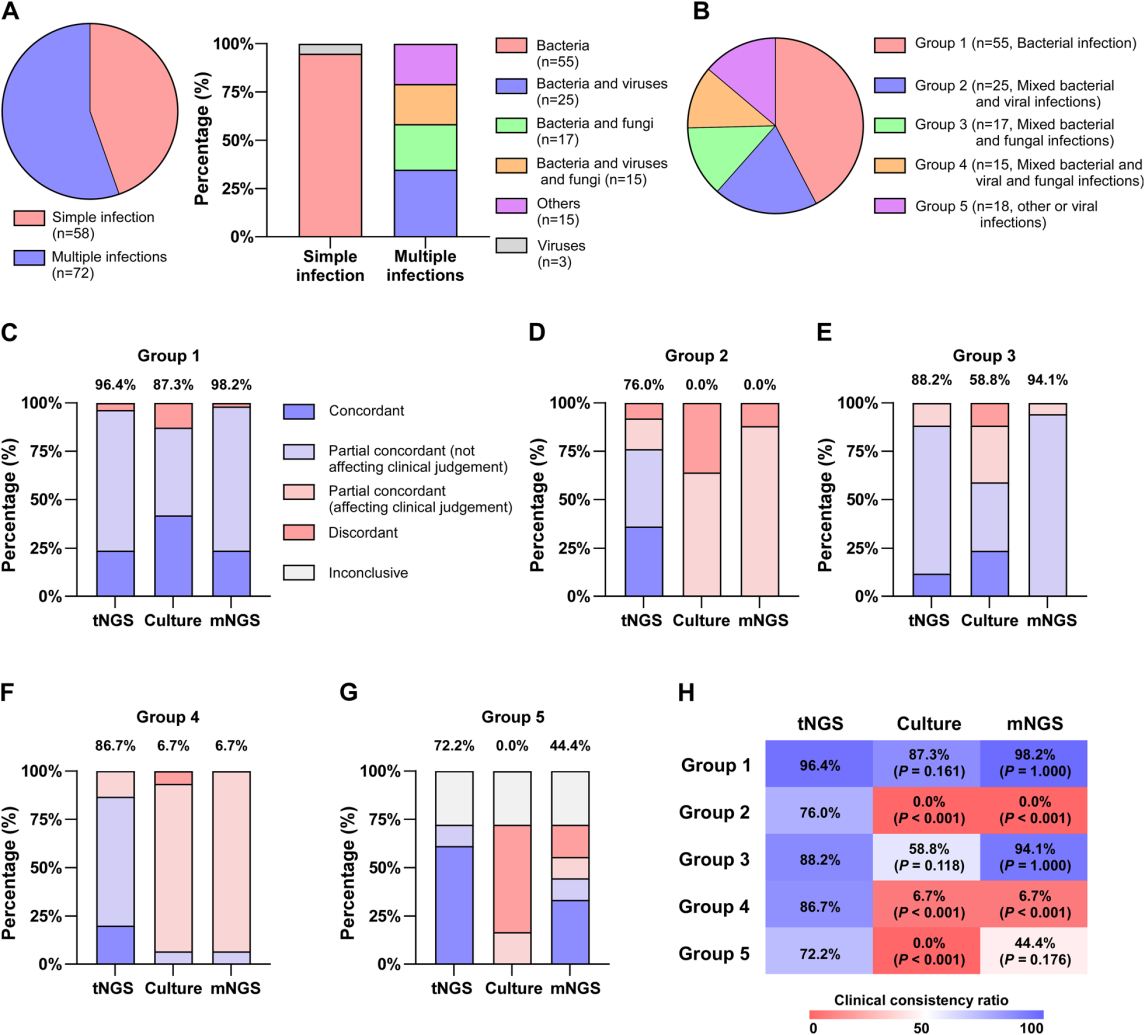
The infection status of 130 patients as well as the evaluation of the concordance between three methods (tNGS, culture, and mNGS) and the clinical diagnostic results. **A** Categorization of pathogenic infections in patients. **B** The subgroup profiles in which the concordance evaluation was conducted. **C**–**G** Evaluation of the concordance of tNGS, culture and mNGS methods with clinical diagnosis in different subgroups. Concordant and partial concordant (not affecting clinical judgement) were deemed to be consistent with the final diagnoses, and the remaining cases were considered incongruous with the final diagnoses. (H) Differences in clinical concordance between tNGS and the other two methods were compared using the χ^2^ test

We evaluated the concordance among the three methods and clinical diagnoses in several subcategories of infections (Fig. 2A, B). In Group 1, all three assays exhibited a clinical concordance of more than 85% (Fig. 2C). The clinical concordance between mNGS and culture was lower than that of tNGS in Groups 2 and 4; the concordance rates of tNGS were 76.0% and 86.7%, respectively (Fig. 2D, F). Groups 3 and 5 exhibited considerably worse clinical culture concordance than did the other methods. The outcomes of tNGS and mNGS were comparable, with tNGS attaining concordance rates of 88.2% and 72.2%, respectively (Fig. 2E, G).

The clinical consistency discrepancy between tNGS and the other methods was assessed. The concordance between tNGS and diagnosis was substantially greater (*P* < 0.001) than that between culture and mNGS in Groups 2 and 4 (Fig. 2H). This investigation was conducted during the COVID-19 and influenza epidemics. Both COVID-19 and influenza viruses belong to the category of RNA viruses; however, only DNA sequencing was used for mNGS. This may have influenced the decreased clinical consistency of mNGS in Groups 2 and 4. Based on the results, tNGS demonstrated excellent clinical diagnostic concordance across all infection types.

### Comparison between tNGS with culture for the detection of common clinical microorganisms

**Fig. 3:**
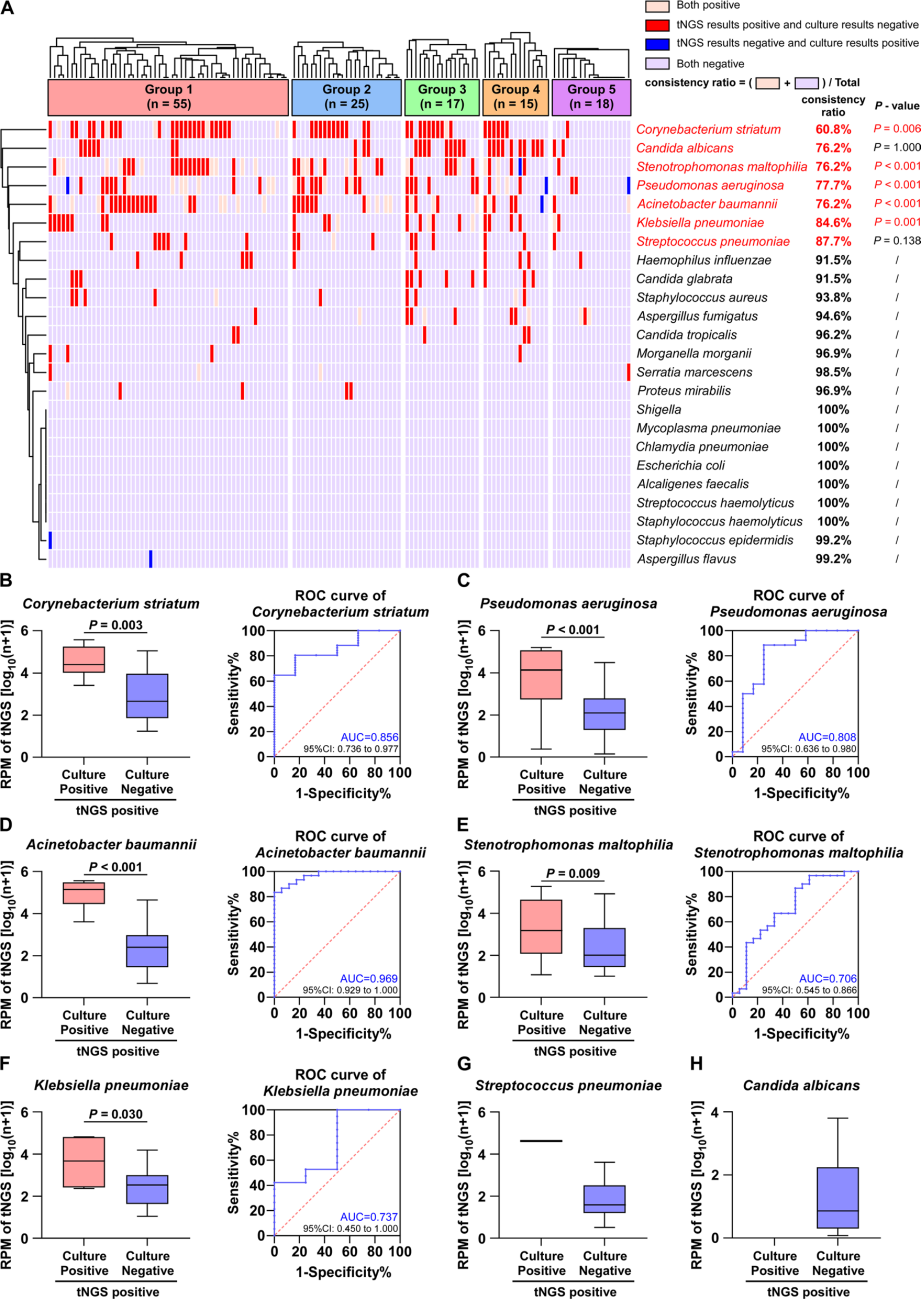
Comparison of tNGS with culture for clinical microorganism detection. **A** Cluster analysis of pathogen detection in tNGS versus culture. **B**–**H** When tNGS detection was positive, it was divided into two groups with positive or negative culture detection and the RPM values of tNGS of the two groups were compared. The ROC curves were plotted using both positive tNGS and culture results as criteria

We selected 24 bacteria or fungi frequently found in clinical diagnoses and conducted a comparative analysis using tNGS and culture techniques. A cluster analysis revealed that almost 70% of the detections made using tNGS and culture had a concordance rate of up to 90%. Most of the disease-causing bacteria, which had a detection concordance of less than 90%, were identified as positive by tNGS but were undetected by culture (tNGS^+^culture^−^) (Fig. 3A).

Additional statistical tests were conducted to investigate the factors contributing to detection rates below 90%. These tests disclosed that RPM values for tNGS were notably higher (*P* < 0.05) in (tNGS^+^culture^+^) samples than for (tNGS^+^culture^−^) samples (Fig. 3B–F). Compared to that for *Corynebacterium striatum*, *Pseudomonas aeruginosa*, and *Acinetobacter baumannii* (AUC > 0.800), there was a lower degree of differentiation for *Stenotrophomonas maltophilia* and *Klebsiella pneumoniae* (AUC < 0.750). For *Streptococcus pneumoniae* and *Candida albicans*, comparisons were not made because of the limited number of samples (Fig. 3G–H). tNGS is, therefore, more sensitive than culture in identifying bacteria and fungi.

### Comparison between tNGS and mNGS for the detection of common clinical microorganisms

**Fig. 4:**
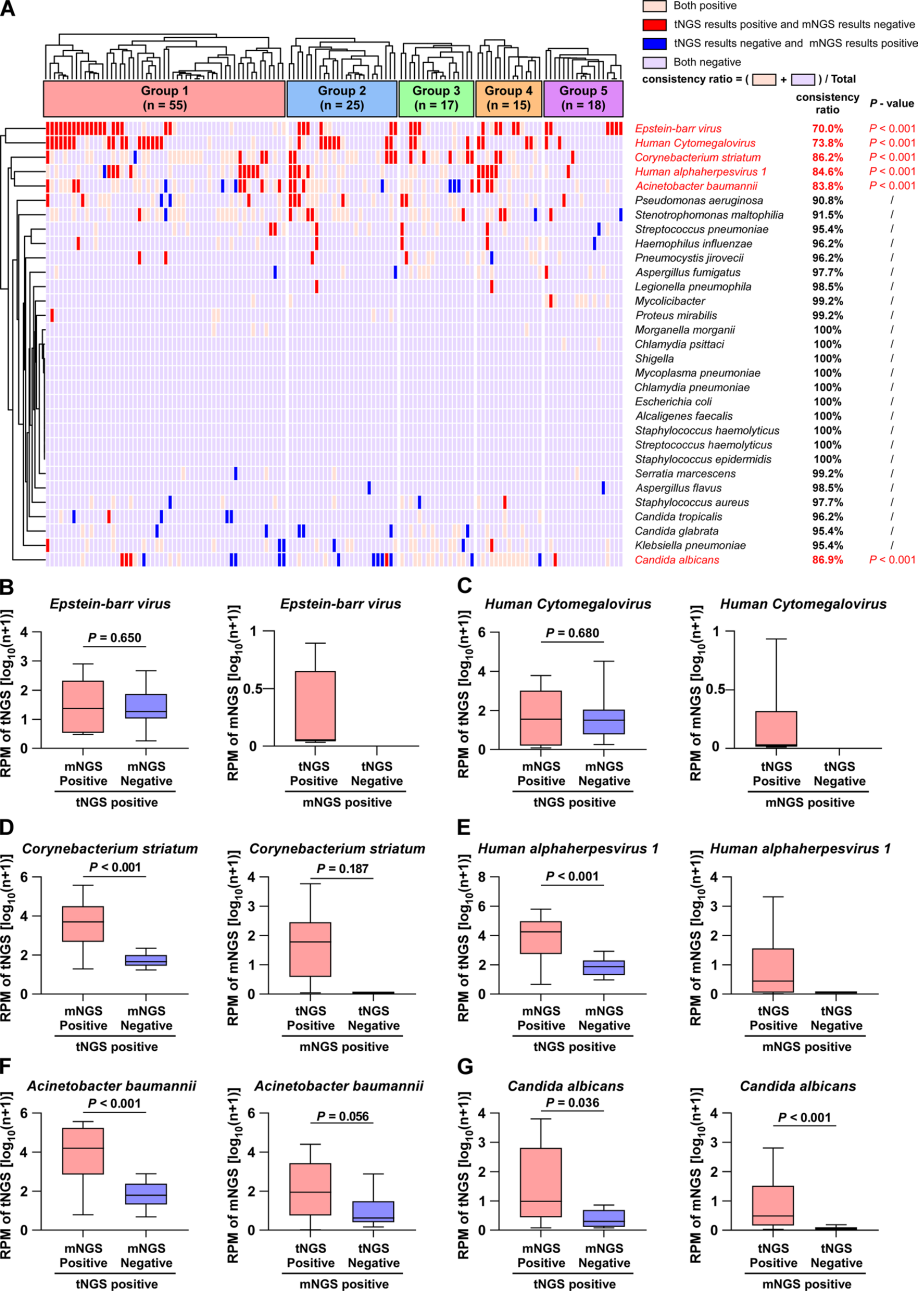
Comparison of tNGS with mNGS for clinical microorganism detection. **A** Cluster analysis of pathogen detection in tNGS versus mNGS. **B**–**G** When tNGS detection was positive, it was divided into two groups with positive or negative mNGS detection, and the RPM values of tNGS of the two groups were compared. In addition, when mNGS was positive, it was categorized into two groups with positive or negative tNGS results, and the RPM values of mNGS in the two groups were compared

We selected 31 microorganisms commonly encountered in clinical diagnoses, including bacteria, fungi, and viruses, and compared their detection results using tNGS and mNGS. A cluster analysis showed that approximately 80% of the tNGS and mNGS detection results had a concordance rate of up to 90%. Pathogenic microorganisms with a detection concordance below 90% were predominantly positive in tNGS and negative in mNGS (tNGS^+^mNGS^−^) (Fig. 4A). Common pathogenic microorganisms, including *Streptococcus pneumoniae*, *Staphylococcus aureus*, *Pseudomonas aeruginosa*, *Acinetobacter baumannii*, and *Klebsiella pneumoniae*, were identified with an accuracy rate of ≥ 83%. In contrast, atypical harmful microorganisms, including *Legionella pneumophila*, *Pneumocystis jirovecii*, *Chlamydia pneumoniae*, and *Mycoplasma pneumoniae*, were detected with a perfect concordance rate of ≥ 95%.

We further investigated the reasons for the detection rates below 90%. For *Epstein-barr virus* (EBV) and *Human Cytomegalovirus* (HCMV), the RPM of tNGS in (tNGS^+^mNGS^+^) samples was not significantly different (*P* > 0.05) from that of (tNGS^+^mNGS^−^) samples. Moreover, there were no cases in which the (tNGS^−^mNGS^+^) samples (Fig. 4B, C). A possible explanation for this may be the low specificity of the probes used to detect EBV and HCMV in the tNGS panel.

For *Corynebacterium striatum*, *Human alphaherpesvirus 1*, and *Acinetobacter baumannii*, the RPM values for tNGS were significantly higher (*P* < 0.001) in (tNGS^+^mNGS^+^) samples than in (tNGS^+^mNGS^−^) samples. Moreover, there was no notable disparity (*P* > 0.05) in the RPM values of mNGS between (tNGS^+^mNGS^+^) samples and (tNGS^−^mNGS^+^) samples. The RPM values of mNGS in (tNGS^−^mNGS^+^) samples were nearly zero (Fig. 4D–F). Additionally, the RPM values of tNGS and mNGS for *Candida albicans* were relatively low, suggesting that tNGS is less effective in detecting *Candida albican*s (Fig. 4G). tNGS and mNGS therefore possess comparable capabilities for identifying harmful microbes; however, their effectiveness in identifying certain viruses needs to be improved.

### Comparison between tNGS and RT-qPCR for the detection of COVID-19 and influenza viruses

**Fig.5:**
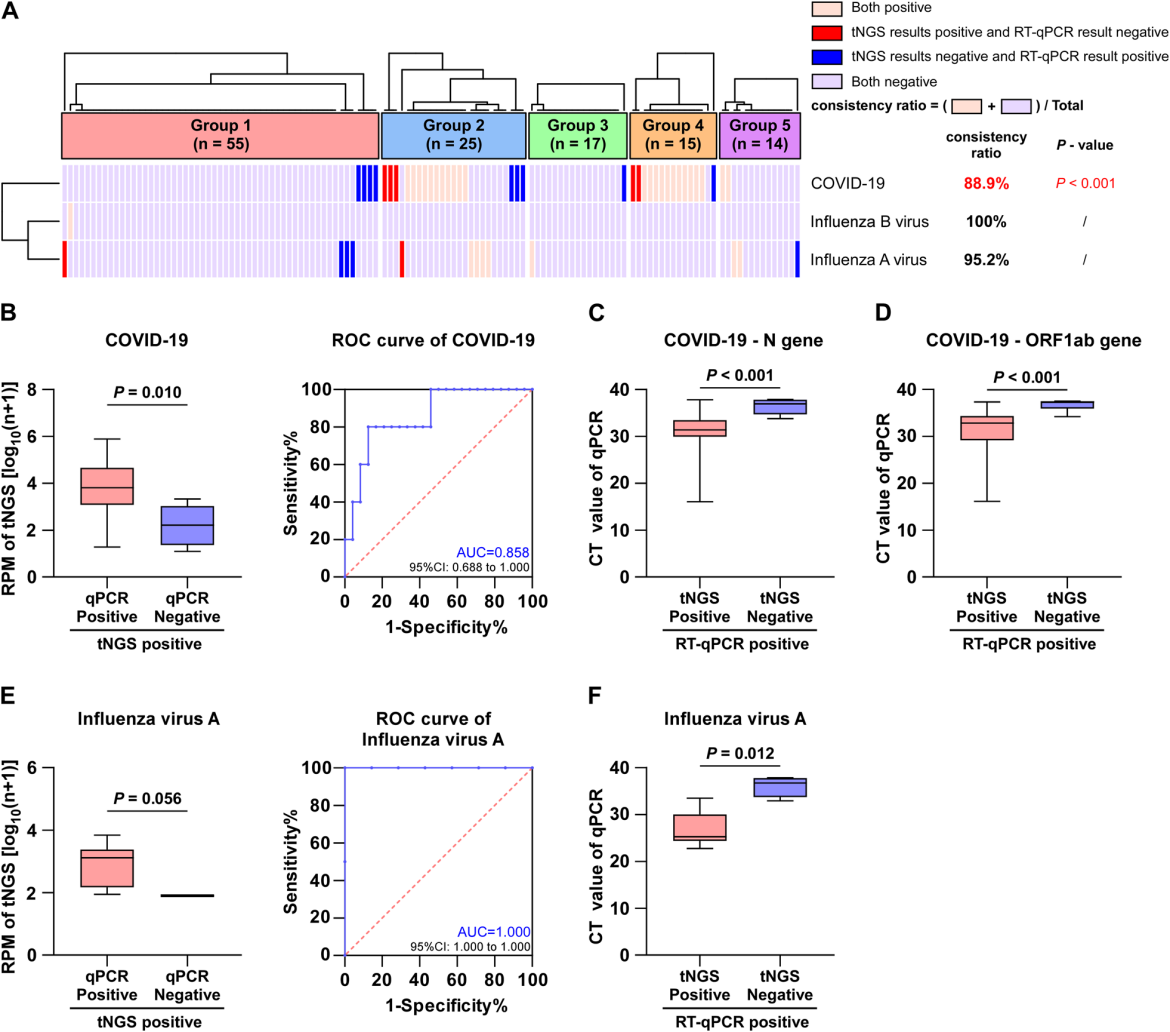
Comparison of tNGS with RT-qPCR for COVID-19, influenza A virus and influenza B virus detections. **A** Cluster analysis of tNGS versus RT-qPCR for COVID-19, influenza A virus and influenza B virus detections. **B** and **E** In COVID-19 and influenza A virus, the RPM values of tNGS in the two groups were compared. The ROC curves were plotted using both positive tNGS and RT-qPCR results as criteria. **C**, **D**, and **F** In COVID-19 and influenza A virus, the CT values of the RT-qPCR of the two groups are compared

To evaluate the efficacy of tNGS in detecting RNA viruses, we compared tNGS and RT-qPCR in detecting COVID-19 and influenza A/B viruses. The concordance between RT-qPCR and tNGS exceeded 88% (Fig. 5A).

For the influenza B virus, the tNGS and RT-qPCR results were consistent. When compared with the COVID-19 detection results, the RPM of tNGS was considerably higher (*P* = 0.010) in (tNGS^+^qPCR^+^) samples than in (tNGS^+^qPCR^−^) samples (Fig. 5B). The CT value of qPCR was also markedly lower (*P* < 0.001) in (tNGS^+^qPCR^+^) samples than in (tNGS^−^qPCR^+^) samples (Fig. 5C–D). The N and ORF1ab genes were the two components of the COVID-19 qPCR detection, and a higher viral load was indicated by a lower CT value. Both tNGS and qPCR therefore exhibited excellent sensitivity. Findings similar to those of COVID-19 were also observed for the influenza A virus. Nevertheless, tNGS showed limited discriminatory ability because there were only two (tNGS^+^qPCR^−^) samples (Fig. 5E–F). Therefore, tNGS is proficient in identifying COVID-19 and influenza A/B viruses, and its detection capability is likely equivalent to that of qPCR.

We also performed a preliminary analysis of COVID-19 viral loads at different locations in 19 cases from the same patient. In approximately 75% of the patients, the CT values of the qPCR for both COVID-19 genes in the swab samples were lower than those of the BALF samples. The viral loads in the swab samples were therefore relatively higher than those in the BALF (Additional file 2: Supplementary Fig. 1).

### An assessment of tNGS for clinical microbial surveillance

To assess the effectiveness of tNGS in monitoring the conditions of critically ill patients, we examined five patients who presented with complicated infections. The outcomes of tNGS microbial semi-quantitative testing were compared with culture results throughout the monitoring of the treatment process. Imaging data and medications for anti-infection therapy were also considered. On average, the tNGS results were consistent with the culture and imaging findings, demonstrating that tNGS is effective in tracking microorganisms during treatments for severe pneumonia. The detailed description of the case is provided in Additional file 3.

To evaluate the results of tNGS resistance detection, three prevalent drug-resistant bacteria were chosen to compare tNGS and culture drug sensitivity results. The concordance between the tNGS and drug sensitivity results was 65.6% for *Pseudomonas aeruginosa*, 68.4% for *Acinetobacter baumannii*, and 65.6% for *Klebsiella pneumoniae* (Additional file 2: Supplementary Fig. 2).

## Discussion

Microbial infections commonly associated with severe pneumonia include those caused by bacterial, fungal, viral, and atypical pathogens [31]. Identifying pathogenic microorganisms is essential for diagnosing and treating severe pneumonia and can enable physicians to develop rational antibiotic treatment plans that reduce drug resistance and enhance infection control. This study found that tNGS results had a concordance rate of > 70% with clinical diagnoses in patients with severe pneumonia. The results of tNGS were consistent with those of culture, mNGS, and RT-qPCR. Several studies have exhibited similar sensitivity and specificity between tNGS and mNGS in different sample types [12, 32]. The tNGS panel used in this study was a primer or probe specifically constructed for 306 pathogens (Additional file 4). We found that tNGS has high detection efficacy for both common and atypical pathogenic microorganisms, and there were no unusual or unique pathogens that were beyond the detection capabilities of tNGS. Furthermore, tNGS has a superior average detection time compared to culture or mNGS. We chose four tuberculosis patients to investigate the efficacy of the tNGS test, and all of them tested positive for *Mycobacterium tuberculosis*. Importantly, tNGS allows the simultaneous acquisition of data on drug resistance genes and pathogen reports. This would enable clinics to promptly identify pathogens and provide information on drug resistance, thereby facilitating treatment guidance.

Nonetheless, this study identified pathogens exhibiting a slightly lower level of concordance (< 90%) with the reference technique. This can be attributed to the technical aspects of tNGS. The enrichment process enhances the minimum detectable level of tNGS, allowing the identification of diseases that cannot be detected using culture or mNGS. Examples include *Acinetobacter baumannii*, *Pseudomonas aeruginosa*, and *Corynebacterium striatum*. Previous studies have also shown that enrichment enhances both the fragment reads of pathogens and coverage of the pathogen genome, thereby aiding accurate pathogen identification [14, 20]. The ability of tNGS to detect a greater number of infections than culture methods may be due to variability in the minimum detection limits among various diseases. Examining tNGS probes targeting *Stenotrophomonas maltophilia* and *Klebsiella pneumoniae* demonstrated that these probes were built with a higher degree of caution than probes designed for other strains specific to these species. The design prioritised genus-level sequences and inadequately covered species-specific sequences, leading to a failure to categorise species-level readings in many instances and a somewhat higher limit of detection. An analogous issue has been reported before [12]. Our data suggest that the minimum detectable level of tNGS for EBV and HCMV is less significant than that for other viruses in comparison with mNGS. The probes used for all viruses encompassed the complete genome and exhibited minimal variation in the tNGS design. Notably, HCMV and EBV can also be detected in the human respiratory system, which may affect patient outcomes [33]. In addition, low *Candida albicans* and *Aspergillus* loads may have been undetected, likely due to the vast genome of the fungus and inadequate probe laydowns. A recent survey also emphasised the necessity of a focused design for tNGS to identify fungi [12].

In this study, five patients with complex infections underwent culture and tNGS semi-quantitative microbiological surveillance. The anti-infection drug prescriptions were modified based on the findings of microbiological tests and additional clinical indicators. tNGS, when used as an enhanced internal reference, is therefore valuable for monitoring pathogenic microorganisms during the treatment of severe pneumonia. Several studies have indicated that adopting qPCR or mNGS techniques to continuously monitor variations in pathogens can be beneficial for understanding patient conditions [34, 35]. When using mNGS to identify pathogens, it is not feasible to evaluate changes in pathogens longitudinally based on the detected reads. Fortunately, the inclusion of specific internal references mitigated the influence of the host and enabled comparisons across samples collected at different timepoints [36, 37].

Numerous studies have explored the benefits of tNGS in detecting drug resistance [26, 38], particularly in tuberculosis resistance [23, 39–41]. tNGS may offer clinicians timely information for the appropriate selection of antimicrobial medicines. Although tNGS is proficient in identifying pertinent drug resistance genes, it is still not capable of substituting for drug sensitivity outcomes.

The major limitation of this study is that the comparison between RNA sequencing using tNGS and mNGS was incomplete because the mNGS assay only uses DNA sequencing. In addition, the duration of this investigation was brief, and the number of samples was restricted. The samples were primarily collected during the COVID-19 and influenza epidemics, leading to minimal detection of DNA virus. Further studies are required to increase the number of samples and integrate prospective and controlled trials, thereby broadening our understanding of the clinical significance of tNGS testing for severe pneumonia.

## Conclusion

Although tNGS is more sensitive than conventional culture for identifying pathogens that cause severe pneumonia, it cannot fully replace drug sensitivity results. In contrast to mNGS, however, tNGS has an analogous capacity to identify clinical microorganisms, making it helpful for clinical efficacy monitoring and guiding choices for anti-infection treatments. Although mNGS has a wider detection range and covers more pathogens, tNGS may become widely employed for clinical infectious diseases as costs continue to drop, because it can account for DNA and RNA pathogens in a single assay, which is more advantageous from a health economics perspective.

### Supplementary Information

Additional file1 (DOCX 371 kb)

Additional file2 (DOCX 1989 kb)

Additional file3 (DOCX 7639 kb)

Additional file4 (XLSX 14 kb)

## Data Availability

The datasets analysed during the current study are available from the corresponding author on reasonable request.

## Abbreviations

ARDS: Acute respiratory distress syndrome
ICU: Intensive care unit
NGS: Next-generation sequencing
mNGS: Metagenomics next-generation sequencing
tNGS: Targeted next-generation sequencing
COVID-19: Corona virus disease 2019
RT-qPCR: Quantitative reverse transcription PCR
BALF: Bronchoalveolar lavage fluid
ROC: Receiver operating characteristic curve
AUC: Area under the curve
EBV: Epstein-barr virus
HCMV: Human cytomegalovirus
