# Supplementary material for: Clinical application of targeted next-generation sequencing in severe pneumonia: a retrospective review
Source: Crit Care. 2024 Jul 8;28:225. doi: 10.1186/s13054-024-05009-8 (PMC11232260; doi:10.1186/s13054-024-05009-8)
Supplement: Supplementary file 1 — Additional file1 (DOCX 371 kb) [file 13054_2024_5009_MOESM1_ESM.docx]

**1 Process of NGS microbiology testing**

1.1 Sample preparation and nucleic acid extraction

The sputum samples were liquefied, and 400 μL was collected. Alternatively, BALF was directly collected. Each sample was independently extracted, resulting in two replicates. Before extraction, 2.6 μL of an internal reference solution was introduced into the 400 μL sample intended for tNGS extraction. The mixtures were thoroughly combined and transferred into a grinding tube, followed by the sequential addition of lysis buffer, Proteinase K mix, and binding buffer. Subsequently, the samples were lysed using a shock crusher. The lysate products were extracted using a VAMNE Magnetic Pathogen DNA/RNA Kit (Novozymes, Nanjing, China). One sample was used for mNGS, and the other was used for RNA reverse transcription using the Hieff NGS® ds-cDNA Synthesis Kit (Yeasen, Shanghai, China) for the tNGS assay.

1.2 Library hybridisation and sequencing analysis

cDNA synthesis and library preparation were performed using the Hieff NGS® C37P4 OnePot cDNA & gDNA Library Prep Kit (Yeasen, Shanghai, China) after extracting all the samples. A copy of the library was prepared and reserved for mNGS. The pre-library was obtained by combining NadPrep® NanoBlockers (Nanodigmbio, Nanjing, China) with tNGS probes (GenePlus, Beijing, China) through hybridisation. The desired pieces were concentrated by immersing the probes in the pre-library for 4 h. The collected products underwent 18 cycles of PCR amplification and were then purified for use. Libraries for online sequencing were generated using a one-step DNB preparation kit. Sequencing was performed using a Gene+Seq-100 sequencer and Gene+Seq-100 High-Throughput Sequencing Kit. Single-end sequencing was used, with a read length of 100 bp. The tNGS sequencing data contained 5 million reads, while the mNGS sequencing data contained 20 million reads. The fastp program was used to conduct basic quality control and filter the data. The filtered reads were matched against a custom-built database of disease-causing bacteria, and the resulting matches were annotated using BLAST software. Reads aligned with the genome of the respective species or the probe-target capture interval were standardised based on the number of reads per million data volumes (RPM). The RPM of each sample of the identified species was compared with that of the corresponding microbes in the negative control, which was tested at the same time in each batch. The finalised detection thresholds were: 5.9 for bacteria, 3.5 for viruses, and 0.5 for fungi and mycobacteria. Samples with RPMs above the threshold were manually validated and reported once the verification process had been completed (Figure below). The tNGS assay lasted approximately 18 h (Fig. 1B), whereas the mNGS assay took approximately 24 h.


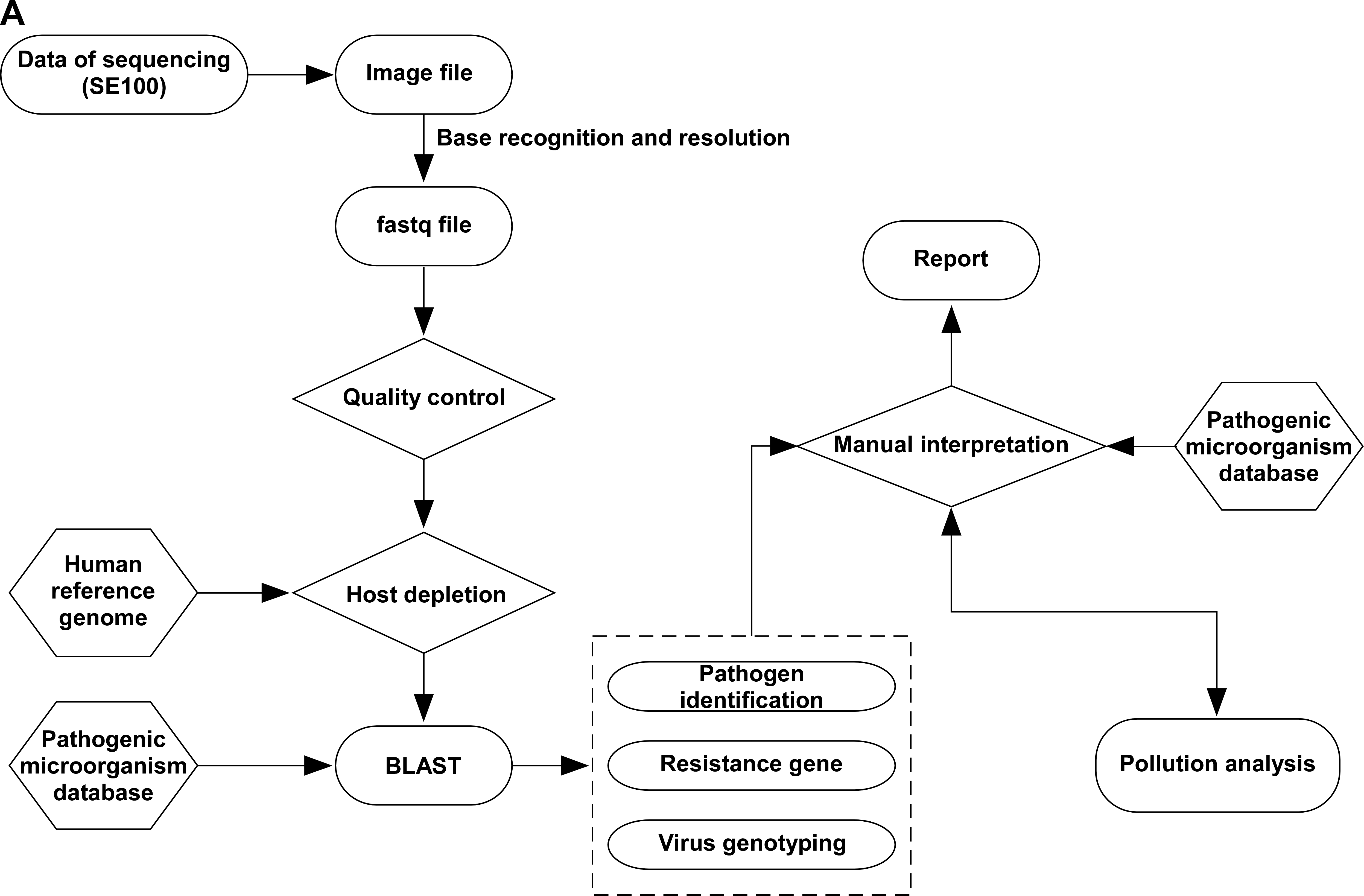


**Figure** Analysis process for tNGS data.

**2 Process of culture microbiology testing**

2.1 sputum samples

On day 1 of the study, sputum samples containing purulent material, sites of blood infection, or digested sputum were collected and placed onto blood agar plates, chocolate agar plates, and MacConkey agar plates for inoculation. The sputum was scraped again and spread onto a slide, and Gram staining was conducted to microscopically assess the quality of the specimens. The inoculated blood and chocolate agar plates were placed into a CO_2_ incubator for 72 h. The MacConkey agar plates were incubated in a thermostatic incubator (35–37 ℃) for 24–48 h. The following day, bacterial species were identified based on the characteristics of the colonies observed in the culture media. Suspicious colonies were directly identified using a VITEK MS mass spectrometer after coating the target plate. It is important to select an appropriate drug sensitivity card for drug sensitivity testing. On day 3, the identification and sensitivity results were documented, verified, and reported.

2.2 BALF samples

After thoroughly mixing, the BALF specimen was vigorously vortexed for 30 to 60 seconds. Following cell plate counting (102 CFU/ml), 10 μL BALF specimens were collected and spotted on blood, chocolate, and MacConkey agar plates respectively. An L-shaped glass rod that had been sterilised was then used to cover the plate. After incubation, the number of colonies is equal to the sum of the colonies with same morphology and the number of colonies at dilution (102 CFU/ml). The cutoff point of 104 CFU/ml was reached by cultures with fewer than 100 colonies. The inoculated blood and chocolate agar plates were placed into a CO2 incubator for 72 h. The MacConkey agar plates were incubated in a thermostatic incubator (35–37 ℃) for 24–48 h. After completing the quantitative culture of the specimen, an appropriate amount of BALF specimen was taken for cell centrifugation, smearing, natural drying, rapid flame fixation for 3 times, and Gram staining. Finally, important pathogenic bacteria of the lower respiratory tract were isolated and identified.

**3 RT-qPCR procedure for COVID-19 and influenza A/B viruses**

The COVID-19 and influenza A/B viruses underwent an analysis using the COVID-19, Flu A and B Multiplex Real-time PCR Kit (Hybribio, Guangzhou, China). The oral pharyngeal swabs were gathered and maintained at 2–8 ℃. The RNA was isolated within 48 h using a kit designed for nucleic acid extraction or purification (Hybribio, Guangzhou, China). Five microlitres of extracted RNA were added to the PCR reaction tubes containing 25 μL of amplification reagent. The tubes were then securely capped and subjected to brief centrifugation. Nucleic acids were amplified and detected by RT-qPCR. A cycle threshold (CT) value ≤ 40 was considered positive.
