# Supplementary material for: Clinical application of targeted next-generation sequencing in severe pneumonia: a retrospective review
Source: Crit Care. 2024 Jul 8;28:225. doi: 10.1186/s13054-024-05009-8 (PMC11232260; doi:10.1186/s13054-024-05009-8)
Supplement: Supplementary file 2 — Additional file2 (DOCX 1989 kb) [file 13054_2024_5009_MOESM2_ESM.docx]

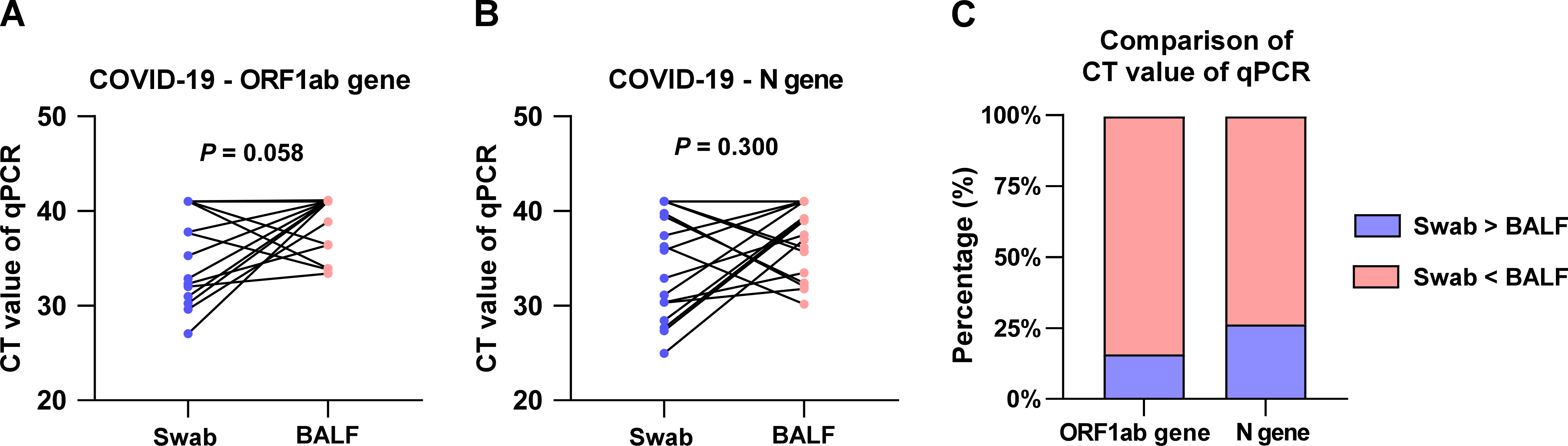


**Supplementary Fig. 1** Comparison of COVID-19 RT-qPCR assays for different samples. (A-B) Pairwise comparative analysis of CT values for RT-qPCR of pharyngeal swabs and BALF (n=19). (C) Most of the CT values of swabs were lower than those of BALF in the assay.


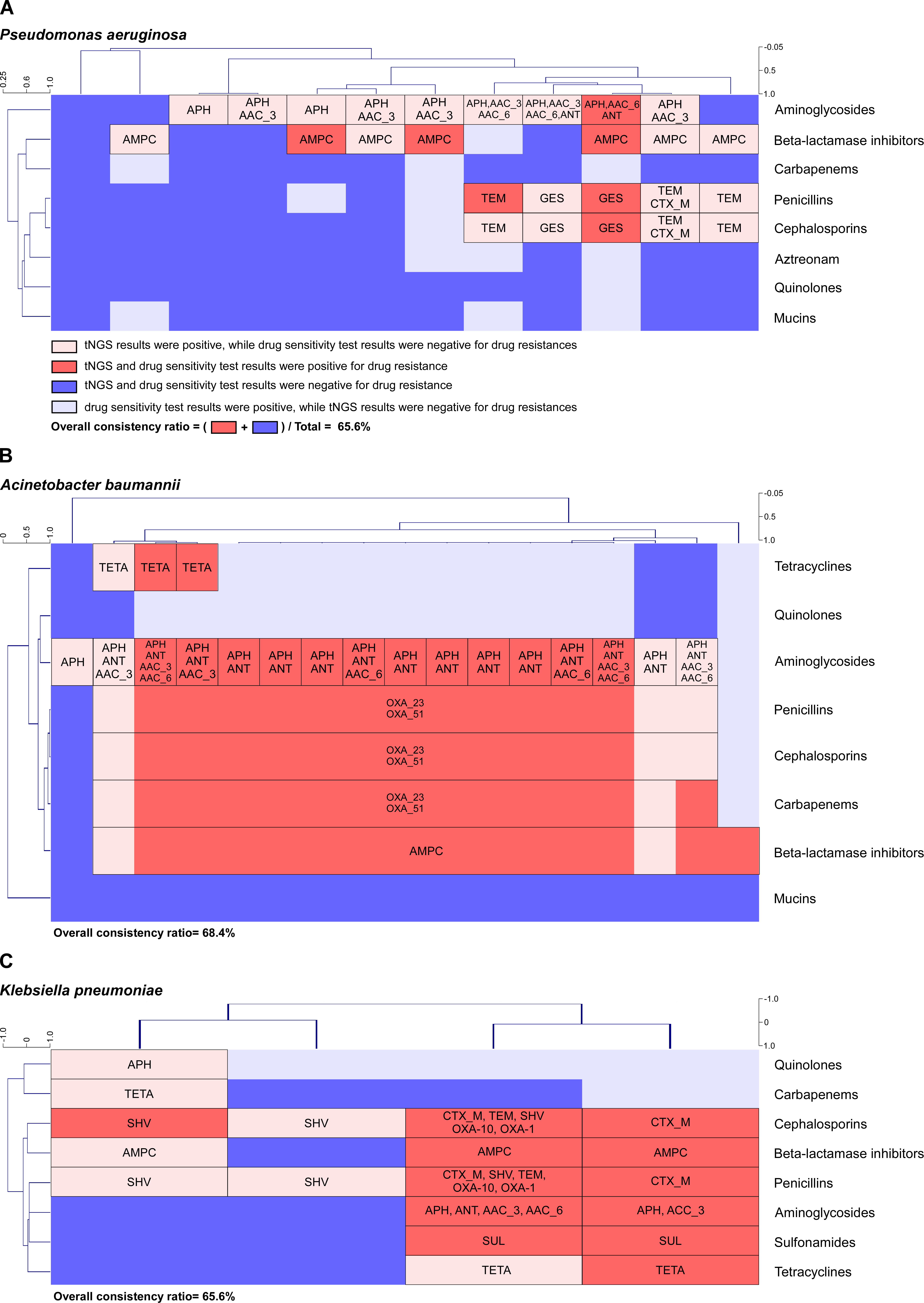
**Supplementary Fig. 2** Analysis of resistance results of tNGS and drug sensitivity tests. (A-C) Cluster analysis of resistance results for *Pseudomonas aeruginosa, Acinetobacter baumannii* and *Klebsiella pneumoniae*. Resistance genes are labelled within the heat map.
