# Supplementary material for: Clinical application of targeted next-generation sequencing in severe pneumonia: a retrospective review
Source: Crit Care. 2024 Jul 8;28:225. doi: 10.1186/s13054-024-05009-8 (PMC11232260; doi:10.1186/s13054-024-05009-8)
Supplement: Supplementary file 3 — Additional file3 (DOCX 7639 kb) [file 13054_2024_5009_MOESM3_ESM.docx]

**Microbiologic monitoring of tNGS in five patients with complicated infections**

Case 1: A 78-year-old male patient received a diagnosis of IFV-A infection along with secondary bacterial infections. The suspected main pathogens were IFV-A and *Acinetobacter baumannii*. On Day 2, cultures showed CR-AB (3+) and *Escherichia coli* (3+). Following the administration of anti-infective medication, the culture findings on Day 8 indicated the eradication of CR-AB and the presence of additional microbes, including *Staphylococcus aureus* (3+), *Corynebacterium* (3+), and *Enterobacteriaceae* (2+). The comparison between Day 2 and Day 8 results revealed a decrease in the RPM values of IFV-A, *Acinetobacter baumannii*, *Escherichia coli*, and *Klebsiella pneumoniae*, while the RPM values of *Corynebacterium striatum* increased. Additionally, new microorganisms such as *Staphylococcus aureus* were detected. These findings indicate an accord between the tNGS and culture outcomes. Imaging revealed a progressive improvement in the exudative lesions in both lungs over time, implying improvement. The patient was eventually extubated on Day 27 and discharged from the ICU on Day 31 (Supplementary Fig. 3A).

Case 2: A 78-year-old male patient received a diagnosis of COVID-19, followed by secondary bacterial infections. The primary pathogen was identified as *Acinetobacter baumannii*. Culture results on Day 9, Day 15, Day 22, and Day 31 showed CR-AB (4+), CR-AB (2+), and CR-AB (1+), and *Serratia marcescens*, respectively. This suggests that CR-AB vanished following the administration of anti-infective treatment to the patient. When the tNGS results were compared to the culture results, the findings were similar: microorganisms like *Serratia marcescens* had emerged, and the RPM values of *Acinetobacter baumannii*, *Corynebacterium striatum*, and *Pseudomonas aeruginosa* had decreased in comparison to the previous values. Imaging showed a gradual improvement of exudative lesions in the right and left upper lungs, suggesting an improvement in the patient's condition. Finally, the patient was taken off the ventilator on Day 35 and transferred out of the ICU on Day 41 (Supplementary Fig. 3B).


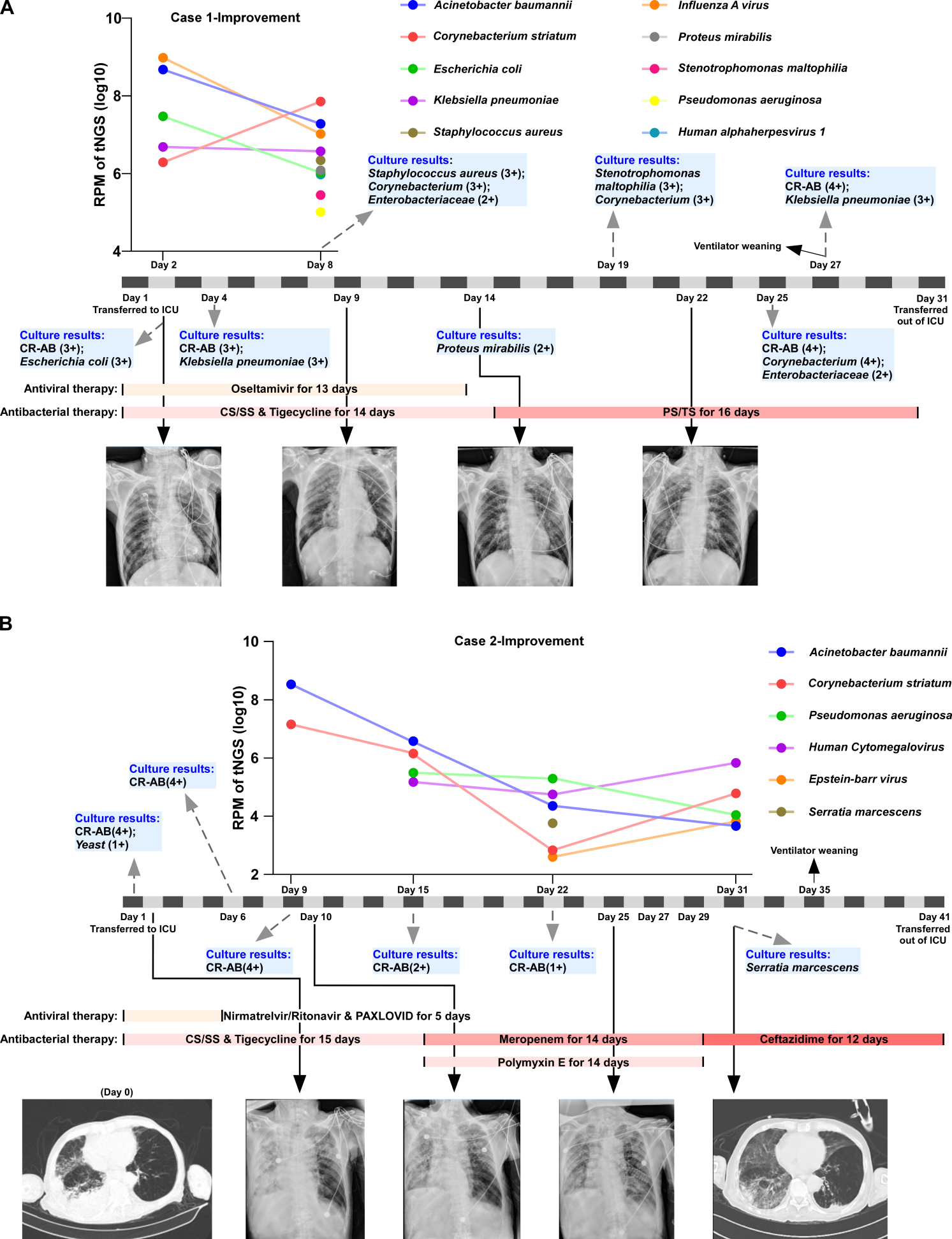


**Supplementary Fig. 3** Schematic timeline of microbiologic testing and treatment profiles of five patients with complicated infections (Case 1 and Case 2). (A-B) Profiles of microbiologic and imaging changes during anti-infective therapy in two patients who improved with treatment. CR-AB, Carbapenem-resistant *Acinetobacter Baumannii*; CS/SS, Cefoperazone Sodium and Sulbactam Sodium; PS/TS, Piperacillin Sodium and Tazobactam Sodium.

Case 3: An 84-year-old man was diagnosed with a COVID-19 infection, followed by secondary bacterial infections. The primary pathogen was probably *Acinetobacter baumannii*. The culture results on Day 2, Day 9, and Day 16 were CR-AB (3+), CR-AB (2+) and CR-AB (3+), respectively. The main pathogenic bacteria identified by tNGS results included *Acinetobacter baumannii*, *Corynebacterium striatum*, *Stenotrophomonas maltophilia* and *Elizabethkingia anophelis*. After receiving anti-infection treatment, the RPM value of tNGS for *Acinetobacter baumannii* showed a decline, but COVID-19 was still detected on Day 9. Later in the treatment, the RPM value of tNGS for *Acinetobacter baumannii* increased again, aligning with the culture results. Possible factors contributing to this situation include the patient's elderly status, compromised lung function resulting from COPD, and the inadequate efficacy of the anti-infective medication. The imaging revealed substantial exudative lesions in both lungs, which did not exhibit remarkable improvement. The patient opted to discontinue medical intervention and was subsequently released from the hospital, ultimately succumbing to their condition (Supplementary Fig. 4A).

Case 4: A 76-year-old male patient was diagnosed with a COVID-19 infection, which was then complicated by secondary bacterial infections. The patient had treatment with VV-ECMO. The primary pathogen was suspected to be *Acinetobacter baumannii*. The culture results showed the presence of *Stenotrophomonas maltophilia* (4+), *Candida glabrata* (3+), and *Enterobacteriaceae* (2+) on Day 1. On Day 10, the culture yielded a negative result. On Day 20, CR-AB (4+) was detected, followed by the detection of *Corynebacterium striatum* (3+) and yeast-like fungi (1+) on Day 28. The tNGS analysis identified the main pathogens, including *Acinetobacter baumannii*, *Corynebacterium striatum*, and *Staphylococcus* species. The RPM values of *Acinetobacter baumannii* and *Corynebacterium striatum* increased from day 10 to day 20, but the culture results became negative by day 28. Despite the improved pathogenetic results, imaging examinations indicated significant exudation and fibrosis in both lungs after contracting COVID-19. Unfortunately, the patient's lung function did not improve, and the treatment proved to be futile, finally leading to the patient's death (Supplementary Fig. 4B).


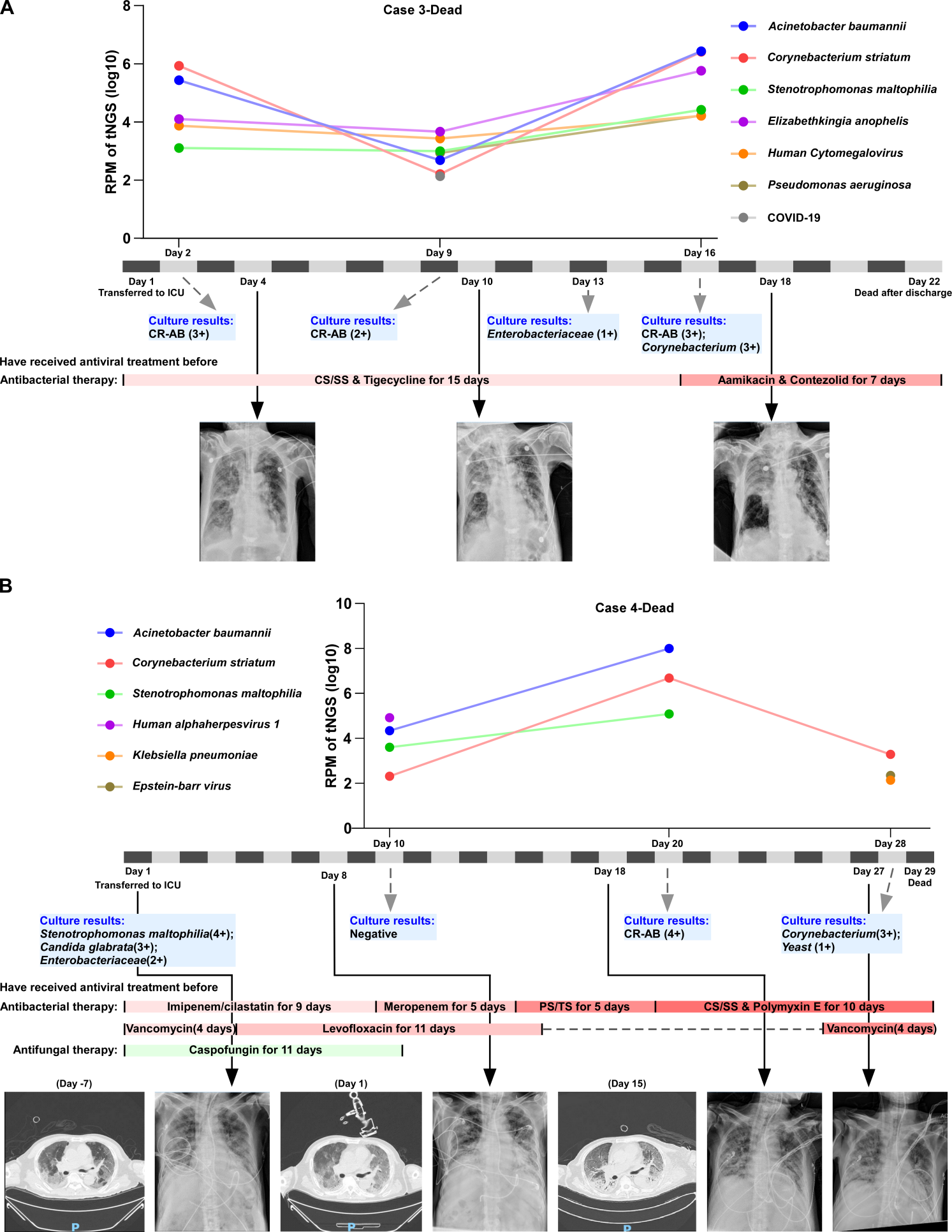


**Supplementary Fig. 4** Schematic timeline of microbiologic testing and treatment profiles of five patients with complicated infections (Case 3 and Case 4). (A-B) Profiles of microbiologic and imaging changes during anti-infective therapy in two patients who eventually died after treatment. CR-AB, Carbapenem-resistant *Acinetobacter Baumannii*; CS/SS, Cefoperazone Sodium and Sulbactam Sodium; PS/TS, Piperacillin Sodium and Tazobactam Sodium.

Case 5: A 59-year-old patient was diagnosed with a COVID-19 infection, subsequently developing secondary bacterial, fungal, and viral infections. The patient received renal transplantation following prolonged use of hormonal and immunosuppressive medications. The main pathogenic microorganisms considered were *Acinetobacter baumannii*, fungi, and COVID-19. The culture results on Day 3, Day 8, Day 18, and Day 32 were negative, CR-AB (4+), CR-AB (1+), and CR-AB (4+), respectively. The main pathogenic microbes found by tNGS include *Acinetobacter baumannii*, *Corynebacterium striatum*, human CMV, COVID-19, and *Pneumocystis jiroveci*. After administering anti-infective medication, the tNGS analysis revealed a decrease in the RPM values of *Acinetobacter baumannii* (Day 10-Day 18), *Corynebacterium striatum* (Day 10-Day 32), human CMV (Day 10-Day 12), and *Pneumocystis japonicus* (Day 18-Day 26). The chest radiographs showed a gradual decrease in exudate from the lower pulmonary regions over 22 days (Day 1-Day 22). Afterwards, the RPM values of tNGS of *Acinetobacter baumannii* increased again as the infection progressed from Day 18 to Day 32, which in accordance the culture results. Subsequently, the Day 35 chest radiograph displayed a heightened presence of exudate in both lungs in comparison to the previous one, ultimately resulting in the patient's death due to treatment failure (Supplementary Fig. 5A). The chest CT scan showed the progressive development of a rounded hollow space in the upper left lung, adjacent to the front of the chest wall, between Day 4 and Day 39. Further, an aggressive tumour was observed growing inside the cavity. The CT scan uncovered clear signs of a lung *Aspergillus* infection, prompting the administration of empirical antifungal drugs. Yet, the presence of *Aspergillus* was not identified through culture or tNGS (Supplementary Fig. 5B).

**
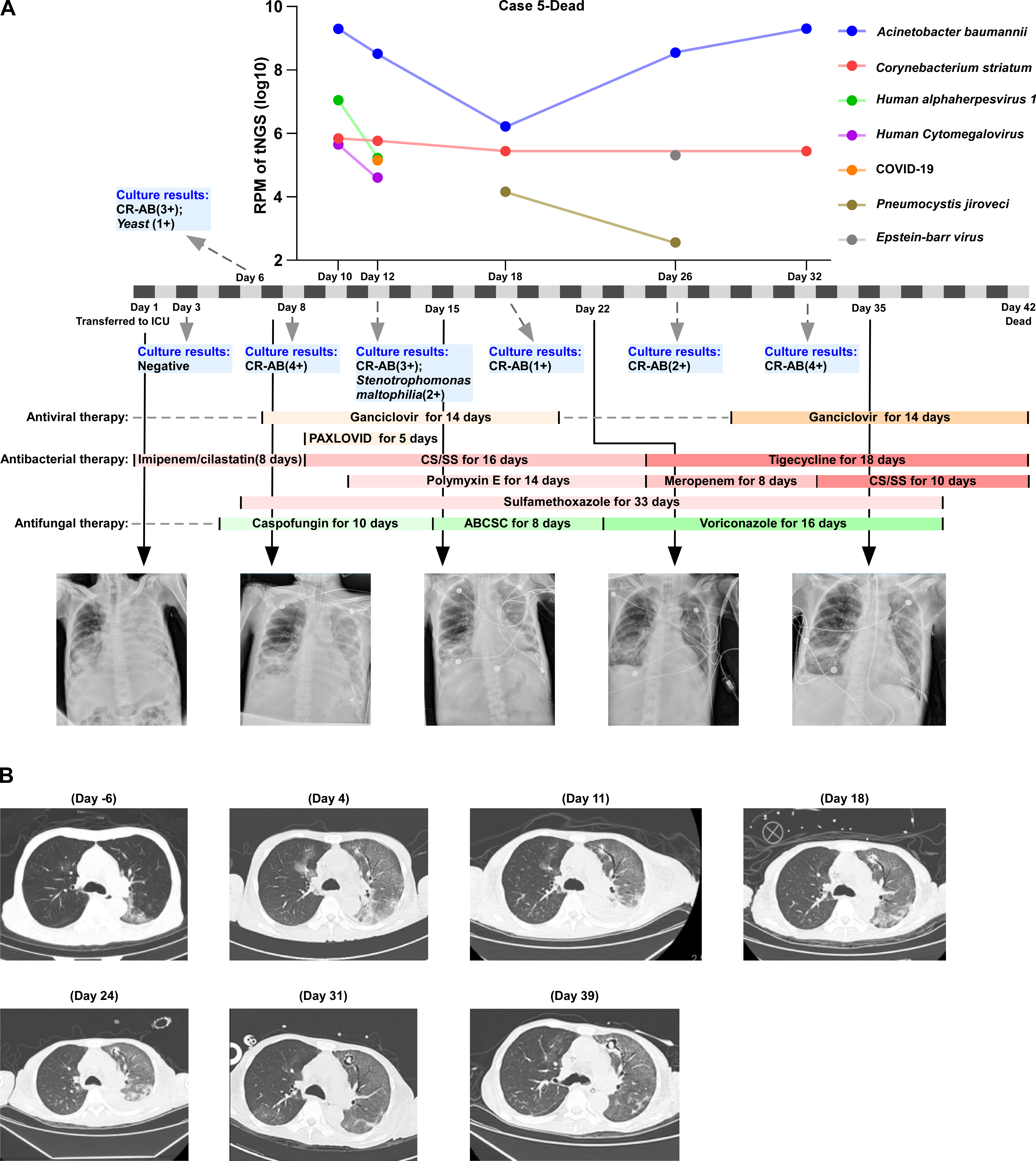
**

**Supplementary Fig. 5** Schematic timeline of microbiologic testing and treatment profiles of five patients with complicated infections (Case 5). (A) Profile of microbiologic and imaging changes during anti-infection therapy in a patient who eventually died after treatment. (B) Complementary imaging profiles of patient at different time points. CR-AB, Carbapenem-resistant *Acinetobacter Baumannii*; CS/SS, Cefoperazone Sodium and Sulbactam Sodium; ABCSC, Amphotericin B Cholesteryl Sulfate Complex for Injection.
